# Cryptococcal Meningitis: A Retrospective Cohort of a Brazilian Reference Hospital in the Post-HAART Era of Universal Access

**DOI:** 10.1155/2018/6512468

**Published:** 2018-08-01

**Authors:** Aline Z. de Azambuja, Gustavo Wissmann Neto, Guilherme Watte, Luciana Antoniolli, Luciano Z. Goldani

**Affiliations:** Section of Infectious Diseases, Hospital de Clínicas de Porto Alegre, Universidade Federal do Rio Grande do Sul, Porto Alegre, RS, Brazil

## Abstract

**Objective:**

*Cryptococcus neoformans* is a common opportunistic infection in adults with acquired immunodeficiency syndrome worldwide. However, limited data exist for HIV-infected patients in the post-HAART (highly active antiretroviral therapy) era in Brazil. The aim of this study was to describe the clinical characteristics and outcomes of cryptococcosis in a cohort of patients attending a teaching tertiary care hospital in southern Brazil after the introduction of HAART in Brazil.

**Patients and Methods:**

A retrospective study was conducted in tertiary care hospital in southern Brazil. Detailed data on risk factors, clinical manifestations, diagnosis methods, treatment, and prognosis of patients with meningeal cryptococcosis were evaluated from January 2009 to December 2016.

**Results:**

Seventy-nine cases of cryptococcal meningitis were identified. Most of the patients presented positive CSF (cerebrospinal fluid) cultures for *Cryptococcus neoformans* (96%). The prevalence of males and females with meningeal cryptococcosis was similar. The age of the patients ranged from 5 to 67 years. The median time of hospitalization was 28 days. The most common underlying disease was HIV (82%), followed by solid transplant (10%). Fever, nausea, vomiting, headache, and altered mental status were the most common clinical manifestations. Initial opening intracranial pressures varied from 30 to 130 cm H_2_O. CNS imaging abnormalities include hydrocephalus and hypodensities. Widened Virchow–Robin spaces were described in only 2 patients (2.5%). Induction treatment of the majority of the patients consisted of amphotericin B and flucytosine (67%) followed by amphotericin B and fluconazole (19%). Multivariate analysis of Cox regression identified headache at presentation, mechanical ventilation, CSF glucose <20 mg/dL, and CSF cryptococcal antigen ≥1 : 1000 for independent risk factors for death. All-cause 30-day and 60-day mortalities were 19% and 24%, respectively.

**Conclusions:**

Meningeal cryptococcosis mostly caused by *C. neoformans* continues to occur predominantly in HIV-infected adults despite HAART being widely distributed in Brazil. Cryptococcosis remains a significant opportunistic infection in solid organ transplant recipients. Despite adequate antifungal treatment and management of intracranial hypertension in a reference tertiary care hospital, mortality was high. Identification of risk factors and additional treatment modalities, especially for intracranial hypertension, are necessary to improve care for patients with cryptococcal meningitis.

## 1. Introduction

Cryptococcal meningitis may be the presenting manifestation of AIDS. The most common sites of occurrence of this infection are the central nervous system and the lungs [1–3]. Inhalation of cryptococcal particles from contaminated soil into the lung is considered the usual route of human infection. This organism disseminates hematogenously, and it has a propensity to localize in the central nervous system, causing meningoencephalitis [2]. Although the incidence of cryptococcal meningitis has declined in the HIV patients who undergo antiretroviral therapy, cryptococcal disease remains a leading cause of mortality in the developing world [4, 5]. The clinical signs and symptoms of *C. neoformans* meningitis are indistinguishable from those of many other causes of meningitis, especially tubercular meningitis. An early diagnosis of the cryptococcal infection is therefore necessary for its appropriate management.

A reduction in the rate of opportunistic infections and hospitalizations in adults infected with AIDS after HAART intervention is well documented in countries with universal access to antiretrovirals [6]. The present work is a retrospective study of patients with cryptococcal meningitis admitted to a Brazilian tertiary care hospital 23 years after the introduction of HAART in Brazil.

## 2. Materials and Methods

This retrospective analysis of epidemiological and clinical data from patients with meningeal cryptococcosis was performed during the period of January 2009 to December 2016 in a tertiary care hospital in southern Brazil. Cryptococcal meningitis was confirmed by a positive CSF cryptococcal culture and/or cryptococcal antigen test. The medical records of all eligible patients were reviewed, and data regarding demographics, medical history, clinical and laboratory characteristics, treatment regimens, and clinical outcomes were collected. Patients were evaluated for risk factors and mortality.

### 2.1. Statistical Analysis

Data were analyzed using SPSS software, version 18 or superior. Results were expressed in mean and standard deviation or median and interquartile range for variables in numeric scale and number and percentage for variables in nominal scale. Qualitative variables were compared by the chi-square test, Fisher's exact test, or McNemar test, when indicated. For determination of the factors associated with overall, 30- and 60-day mortalities, multivariate logistic regression models were constructed using a stepwise regression. A predictive modeling strategy was used in which variables were selected based on the association with mortality in univariate analysis (using a *p* value ≤ 0.20) and retained if they significantly improved model fit. The Cox regression model was used for determination of risk factors associated with overall mortality. A Poisson regression model with robust variance was employed for determination of risk factors associated with mortality at 30 and 60 days. The Kaplan–Meier survival method was adopted for survival analysis of cohort. The survival between groups (HIV and non-HIV) was compared by log-rank test. For statistical significance, a *p* value ≤ 0.05 was considered.

## 3. Results

As shown in Table 1, seventy-nine cases of cryptococcal meningitis were identified. Most of the patients presented positive CSF cultures for *Cryptococcus neoformans* (96%). The prevalence of males and females with meningeal cryptococcosis was similar. The age of the patients ranged from 5 to 67 years. As expected, the mean time of hospitalization was long (28 days). The most common underlying disease was HIV (82%), followed by solid transplant (10%). Only 34 (43%) patients were on highly active antiretroviral therapy (HAART). Medication adherence was not measured in these patients. The vast majority of the patients with meningeal cryptococcosis were infected by *Cryptococcus neoformans* (96%). Fever, nausea, vomiting, headache, and altered mental status were the most common manifestations (Table 2). Laboratory findings are described in Table 3. High CSF cryptococcal antigen was present in 60% of the patients. The majority of the patients developed intracranial hypertension. Initial opening Intracranial pressures varied from 30 to 130 cm H_2_O. Despite appropriate antifungal treatment, patients persisted with intracranial hypertension. The CNS imaging abnormalities include hydrocephalus and hypodensities. The classical finding of widened Virchow–Robin spaces was described in only 2 patients (2.5%). Induction treatment of the majority of the patients consisted of amphotericin B and flucytosine (67%) followed by amphotericin B and fluconazole (19%) (Table 4). Fluconazole was combined with amphotericin B for some patients because flucytosine was not commercially available in Brazil for a certain period of time. All-cause overall 30-day and 60-day mortalities were 19% and 24%, respectively. Multivariate analysis of Cox regression identified headache at presentation, mechanical ventilation, CSF glucose <20 mg/dL, and CSF cryptococcal antigen ≥1 : 1000 as independent risk factors for mortality.

## 4. Discussion

The incidence of cryptococcal meningitis has increased after the pandemic of AIDS and despite availability of HAART continues to be the most common cause of opportunistic meningitis in many countries [4–6]. Although the number of cases of cryptococcal meningitis diminished in the HAART era, Brazil, Colombia, Mexico, and Argentina are the countries with the highest incidence of meningeal cryptococcosis in Latin America [7]. Although access to HIV diagnosis, universal access, and immediate start to HAART therapy constitutes a major medical advancement in the clinical management of HIV in Latin American countries such as Brazil, its success depends on strict adherence to prescribed regimen [8]. Approximately 50% of our patients with meningeal cryptococcosis were on HAART, with probably poor adherence to medical treatment. In a recent study in Brazil, a higher frequency of patients with CD4 + cell count <200 cells/mL was observed in 2014-2015 when compared to the previous years [9]. This suggests that the adopted strategies for early diagnosis of HIV/AIDS are still ineffective. In contrast, with the advent of effective antiretroviral therapy, the majority of cases of cryptococcal meningitis in developed nations occur among non-HIV-infected patients, especially organ transplant recipients [10]. In fact, cryptococcosis is the third most commonly occurring invasive fungal infection, after invasive candidiasis and aspergillosis, in solid organ transplant recipients [11]. It has been observed that cryptococcal meningitis in liver and kidney recipients are late infections in our institution, and considered to represent reactivation of latent infection in the recipient. The median time to cryptococcal disease from transplantation in our study was 382 days (data not shown).

As expected, *Cryptococcus neoformans* species complex was the predominant species isolated in our patients. *C. neoformans* is globally the major fungal pathogen causing infections in immunocompromised individuals such as AIDS patients, with an estimated incidence of 223,100 cases in 2014 [4]. *C. neoformans* has long been considered as the culprit causing cryptococcosis in HIV-infected patients including countries in Latin America [12, 13]. The third largest number of cases in the world occurred in Latin America, with an estimated incidence of 5,300 cases per year. Brazil and Colombia followed by Argentina and Mexico were the countries with the highest incidence [4].

Despite availability of universal HAART, appropriate antifungal treatment including amphotericin B and flucytosine/fluconazole, and management of intracranial hypertension in a reference tertiary care hospital according to the IDSA guidelines, our mortality was high (60-day mortality of 25%) [14]. The median time between diagnosis of cryptococcal meningitis and HAART initiation was 21 days (data not shown). In our institution, we recommend that HAART initiation should be delayed until there is evidence of a sustained clinical response to antifungal therapy. Early initiation of HAART resulting in IRIS has been described in patients with tuberculous meningitis, and the evidence remains mixed for those with cryptococcosis [15]. Additional studies are warranted to provide the evidence base for effective decision making. Considering our approach of delayed initiation of HAART, we observed only five cases of immune reconstitution inflammatory syndrome which did not impact in the interpretation of our results (data not shown).

Cerebrospinal fluid high opening pressure occurred in the majority of our patients and persisted despite adequate antifungal treatment. In fact, the kinetics of release of a pivotal cytokine like TNF-a in immune responses induced by cryptococcal cell envelope components will reflect on the course of the infection, its containment, and ultimately its elimination by phagocytes [16, 17]. Headache was an independent risk factor for mortality in our study and considered the most common symptom of intracranial hypertension. In addition, CSF glucose levels <20 mg/dL and cryptococcal antigen ≥1 : 1000 was identified as an important risk factor for death in our cohort. Previous studies have shown that initial high CSF cryptococcal antigen titers were independent predictors of mortality in cryptococcal meningitis, especially in non-HIV-infected patients [18, 19]. Considering Brazil as a middle-income country by the World Bank (2017), our mortality was lower than the estimated mortality for middle-income countries and similar to other countries in Europe and North America. Rajasingham et al. estimated 1-year mortality in low-income countries as 70% after cryptococcal meningitis for those in care and 100% for those not in care. In middle-income countries, the estimated 1-year mortality was 40% (uncertainty interval 34–46), based on summary statistics of outcomes of those receiving amphotericin B and fluconazole, and 60% for those not originally in care [4]. In Europe, the estimated 1-year mortality was 30% for those in care and 45% for those not in care. In North America, the estimated 1-year mortality was 20% for those in care and 30% for those not in care [4].

In summary, the present study highlights important characteristics of a recent cohort of patients with cryptococcal meningitis attending a tertiary care hospital in Brazil after 23 years of the institution of universal HAART. Cryptococcal meningitis continues to be a prevalent opportunistic infection in HIV-infected patients. In addition, mortality and adverse neurologic sequelae from HIV-associated cryptococcal meningitis remain high due to raised intracranial pressure complications despite appropriate treatment. Identification of risk factors and additional treatment modalities, especially for intracranial hypertension, are necessary to improve care for patients with cryptococcal meningitis. Although cryptococcal disease rates are decreasing over time, the associated mortality and costs remain concerning.

## Figures and Tables

**Table 1:** Baseline characteristics of 79 patients with cryptococcal meningitis.

Variable	
Age, years; mean (range)	37 (5–67)
Age ≥50 years; *N* (%)	17 (21.5)
Sex, male *N* (%)	45 (56.9)
Time of hospitalization,days; mean (range)	28 (1–149)
*Type of immunosuppression*	*N* (%)
HIV	65 (82.3)
SOTR	8 (10.1)
NHNT	6 (7.6)
*HIV patients*	
Dx during hospitalization^a^	14 (17.7)
Vertical transmission of HIV	3 (3.7)
Receiving HAART at Dx	34 (43.0)
CD4, cells/mm³ mean (range)	34 (3–428)
HIV viral load, log; mean (range)	5.01 (2.18–6.16)
*SOTR patients*	*N* (%)
Kidney	5 (10.1)
Liver	3 (10.1)
*Duration of transplant, years*	
General; mean (range)	2.12 (0–7.35)
Kidney; mean (range)	3.58 (0.25–7.36)
Liver; mean (range)	2.12 (0–7.35)
*Other concomitante conditions*	*N* (%)
Use of corticosteroids	8 (10.1)
Chronic kidney disease	2 (2.5)
Cancer	4 (5.1)
Rheumatic disease	2 (2.5)
Diabetes mellitus	3 (3.8)
Cirrhosis	2 (2.5)
*Cryptococcus species complex*	
*C. neoformans*	76 (96.2)
*C. gattii*	3 (3.8)
Duration of symptoms, days; mean (range)	10 (1–120)
Rehospitalization	6 (7.5)
30-day mortality	15 (19.1)
60-day mortality	19 (24.4)

SOTR, solid organ transplant recipients; NHNT, non-HIV nontransplant; ESRD, end-stage renal disease. ^a^Diagnosis of HIV concomitant with neurocryptococcosis.

**Table 2:** Clinical manifestations of 79 patients with cryptococcal meningitis.

Variable	*N* (%)
Fever	43 (54.4)
Malaise	22 (27.8)
Headache	58 (73.4)
Nausea and vomiting	33 (41.8)
Altered mental status	34 (43.0)
Visual disturbances	8 (10.1)
Behavior changes	7 (8.9)
Seizures	11 (13.9)
Nuchal rigidity	15 (18.9)
Cranial nerve palsy	8 (10.1)
Motor deficit	8 (10.1)
Cough	19 (24.1)
Dyspnea	9 (11.4)
Constitutional symptoms	21 (26.6)

**Table 3:** Laboratory findings of 79 patients with cryptococcal meningitis.

Variable	Initial	^a^After treatment	*p* value
	Mean (range)	Mean (range)	
Opening pressure (cm H_2_O)	30 (10–130)	22 (10–50)	0.387
CSF glucose (mg/dL)	36.5 (1–145)	43 (2–154)	0.278
CSF leukocytes (cells/*µ*L)	25 (1–853)	8 (0–300)	<0.05
CSF proteins (mg/dL)	82 (0–882)	59 (0–1866)	0.228

	(%)	*N* (%)	

CSF antigen^b^ ≥1 : 1000	(55.6)	7 (8.8)	0.642
CSF positive culture	(93.6)	6 (7.5)	1.00
Blood culture positive^c^	(45.5)	—	—
Blood antigen ≥1 : 1000^b^	(40.5)	—	—
Bronchoalveolar lavage positive	(7.5)	—	—

CSF, cerebrospinal fluid. ^a^After 2-week course of induction therapy. ^b^Cryptococcus antigen. ^c^Collected within 14-day period of induction therapy.

**Table 4:** Comparison between induction treatment schemes of 79 patients with cryptococcal meningitis.

Variable	AmB + 5-FC	AmB + flu	*p* value
	Mean (range)	Mean (range)	
Time, weeks	2.65 (0.57–14.4)	2.6 (1–5.43)	—
AmBd dose^a^	50 (20.6–69.1)	52.4 (50–90)	—
LFAmB dose	200 (100–371)	275 (200–375)	—
5-FC dose	6000 (1280–9800)	—	—
Flu dose	—	800 (300–1000)	—
	(%)	(%)	
Hypokalemia	(67.0)	(15.1)	—
Hypomagnesemia	(67.0)	(8.8)	—
Acute renal injury	(67.0)	(10.1)	—
Anemia	(67.0)	(12.6)	—
Flu-consolidation	(51.8)	(15.1)	—
Time of consolidation, weeks	8 (1.5–8)	7.8 (2–8)	—
30-day mortality	(11.4)	(26.7)	0.210
60-day mortality	(17.4)	(26.7)	0.462

AmB, amphotericin B; 5-FC, flucytosine (mg/day); Flu, fluconazole (mg/day); IQR, interquartile range; AmBd, amphotericin deoxycholate; LFAmb, lipid formulations of amphotericin B. ^a^Dose reported in mg/day.

## Data Availability

The data used to support the findings of this study are available from the corresponding author upon request.
